# Understanding the audience in framing research: empirical evidence from three studies examining HIV framing in China

**DOI:** 10.3389/fpubh.2023.1172020

**Published:** 2023-08-17

**Authors:** Tianen Chen, Minhao Dai, Nancy Grant Harrington

**Affiliations:** ^1^Department of Communication, Cornell University, Ithaca, NY, United States; ^2^School of Communication and Media, Kennesaw State University, Kennesaw, GA, United States; ^3^Department of Communication, University of Kentucky, Lexington, KY, United States

**Keywords:** HIV framing, Chinese government-sponsored newspapers, political environment, socio-cultural impacts, mixed methods

## Abstract

Guided by framing theory, this three-phase mixed-methods study explored (a) how Chinese government-sponsored newspapers frame HIV and (b) framing effects on people’s HIV beliefs. A content analysis of two government-sponsored newspapers and a survey of 210 readers showed discrepancies in frame and frame valence. In-depth follow-up interviews with 15 media and public health experts revealed that the discrepancies were related to people’s attitudes toward the media and beliefs about HIV, which could further be explained by the political environment, media ecology, historical framing, and cultural identities in China. We discuss theoretical implications for framing theory and practical implications for HIV media coverage.

## 1. Introduction

China has been extending its influence on the world’s media, and Chinese media occupy an important and powerful place in the global media landscape (1). Chinese media are unique because of China’s complex power discourse and cultural characteristics (2, 3). For example, most traditional media in China, such as newspapers and magazines, are directly or indirectly owned by the central government (4). Government-sponsored newspapers (GSNs) are granted a very limited degree of freedom and only cover news issues that are considered to be “appropriate” (5), maintaining informational hegemony. Being positive and constructive are two of the key characteristics of Chinese-produced news content (6). GSNs such as *People’s Daily* serve as the mouthpiece of the Communist Party of China, provide information about “official” points of view, and set a frame of reference and the tone of coverage for other media across China (7).

Given GSNs’ unique positions and powerful influences, it is necessary for scholars to explore and investigate Chinese media content and environment, as well as the media’s impacts on Chinese people’s beliefs and attitudes. Thus, the current study applies framing theory (8, 9) to explore how Chinese GSNs framed human immunodeficiency virus (HIV) and how well such framing has influenced Chinese readers’ HIV beliefs through a three-phase mixed-methods study. HIV was chosen as the contextual topic because it is a prevalent infectious illness in China with severe public health consequences (10) and also because social policies related to HIV are gradually changing in China. A series of HIV-related policies and social changes (e.g., legislation protecting the rights of people living with HIV) have been implemented in China (11), and the news coverage of HIV is changing, as well. Thus, examining the framing of HIV and its effects in China is not only an interesting case study for media framing research but also could help people understand this important public health issue better.

## 2. Literature review

### 2.1. Framing theory

Framing has two foundations: sociological and psychological (12). Framing research from the sociological foundation suggests that people rely on socially constructed frameworks to process and classify new information and interpret issues (13). This sociological approach focuses on emphasizing certain facts over others and has been referred to as emphasis framing (14). From the sociological perspective, a media frame can be defined as “the central organizing idea for news content that supplies a context and suggests what the issue is through the use of selection, emphasis, exclusion, and elaboration” (15). Framing research from the psychological foundation focuses on how media frames influence the formation of audience frames and the subsequent consequences [e.g., decision making, preference (12)]. Psychologically rooted framing has been referred to as equivalency framing because it deals with the variation or manipulation of the same piece of information (14, 16). More specifically, this type of research investigates how different presentations of the same message can invoke different interpretive schemas among audiences and lead to different interpretations of the message (13).

Scheufele (17) developed a model of framing that includes four processes: frame building, frame setting, individual-level effects of audience frames, and journalists and elites as audiences. Frame building deals with what and how external factors (e.g., elites and authorities), organizational-level factors (e.g., the political orientation of a news agency), and journalist-centered factors (e.g., professional norms and ideology) influence the formation of frames in news and media; frame setting captures the process in which media frames influence the formation of audience frames; individual-level effects of audience frames refer to the link between audience frames and individual-level outcomes (e.g., attitudes and behavior); lastly, journalists and elites, as audiences and cognitive misers, may be influenced by frames (17).

The current study examines the frame setting in which media frames influence the formation of audience frames. Successful news frame setting has been well documented by previous research across various contexts. For example, de Vreese (18) investigated the effects of TV news frames on people’s interpretation of a political issue related to the European Union’s development. The results indicated that news employing a conflict frame caused more conflict-related thoughts in participants and that news employing an economic consequences frame caused more thoughts related to economic consequences. Dhanani and Franz (19) compared three ways of framing COVID-19 information: the origin of COVID-19, economic harm, and health threat. The results indicated that frames that emphasized the origin of COVID-19 (China) increased negative and xenophobic attitudes toward Asian Americans. In health contexts, Coleman et al. (20) found that health news stories with a thematic frame (e.g., attributing the responsibility for causing obesity to societal-level factors such as limited access to healthy food and exercise) were more likely than those with an episodic frame (e.g., attributing the responsibility for causing obesity to individuals) to increase people’s intention to change their health behaviors. Riles et al. (21) investigated the effects of three news frames (lifestyle, environmental, and medical) on people’s perceptions of cancer and cancer patients. The lifestyle frame attributes the responsibility for getting cancer to patients and their lifestyles; exposure to that frame was associated with more thoughts about cancer risk factors, stigma, and blame than exposure to the environmental and medical frames. More recently, in the context of COVID-19 vaccination promotion, Palm et al. (22) found that frames that emphasize vaccine safety and efficacy positively predicted people’s intention to get vaccinated against COVID-19.

In addition to the impacts related to the content of the frame, previous studies also suggested that the frame’s valence plays a role in influencing people’s opinions, thoughts, and emotions. For instance, Lecheler et al. (23) found that news frames had affective power on people’s opinions about immigration: Positive frames caused more positive opinions, and negative frames resulted in more negative opinions. Fernández et al. (24) found that participants were more likely to use negative affective language to express their thoughts on immigration after exposure to news stories using negative frames than those exposed to news stories with positive frames.

### 2.2. Framing of HIV

Historically, media presented HIV issues through frames of victimization, deviance and abnormality, denial, and blame attribution and the early coverage of HIV/AIDS focused on the deviant behavior or character of “victims” instead of the disease itself (25). Media coverage in the 1980s and 1990s often labeled AIDS as a disease of the “other” and blamed homosexual populations, sex workers, drug users, and other marginalized groups for the spread of HIV (26, 27). Recent media coverage of HIV/AIDS seems to have moved away from the blame attribution and deviance approaches. Kiwanuka-Tondo et al. (28) content-analyzed 365 articles related to HIV and AIDS from major newspapers in Uganda. The study results indicated that the “curative medicine” and “HIV infection rates” frames received the most coverage, whereas “victim” and “higher-risk behavior” frames were less utilized. de Souza (29) performed a comprehensive analysis of HIV-related news articles from India and found that journalists often employed three types of frames when reporting HIV-related issues: severity, causes and solutions, and beliefs about who is at risk. The results of these two studies suggest that media coverage of HIV has started to devote attention to the disease itself. However, Goh (30) conducted a textual analysis of articles from a major newspaper in Singapore and found that these news articles still framed homosexuality as promiscuous and assigned the blame to the gay population for the rise of HIV in Singapore. Kiptinness and Kiwanuka-Tondo (31) content-analyzed HIV-related newspaper articles from Kenya and found that these articles frequently used victim framing (depicting people living with HIV as victims and highlighting their need of help). Kiran and Mahmood (32) analyzed articles from two major newspapers in Pakistan, and the study results indicated that the majority of the articles used a “disaster” frame.

### 2.3. Framing of HIV in Chinese media

There have been several studies on Chinese media coverage of HIV issues in recent years. For example, Gao et al. (33) examined the coverage of HIV/AIDS in Chinese newspapers from 2000 to 2010 and found that newspaper articles (*n* = 3,648) paid most attention to prevention, followed by treatment, education and awareness, clinical science, statistics, transmission and risk, policy and economics, and people living with HIV. Among the 3,648 articles analyzed, 299 articles had a negative valence, 2,451 had a neutral valence, and 898 had a positive valence. In another example, Wu (34) conducted a framing analysis of the news coverage of HIV/AIDS from Xinhua News Agency (the official party news organization in China); the results indicated that most of the coverage was devoted to the strategies that the government has taken in promoting and developing HIV/AIDS prevention methods and medical treatments. Wu suggested that Xinhua News Agency’s coverage of HIV issues was mostly along the party line: emphasizing the government’s commitment to protecting its people and its competence in dealing with HIV issues. Tong (35) compared the coverage of HIV/AIDS in *The New York Times* and *China Daily* between 2001 and 2004 through the lens of framing. Tong suggested that compared to *The New York Times, China Daily* applied a milder tone when describing HIV issues, frequently constructed a positive image of the government in handling HIV issues, and focused more on prevention and social problems. Such findings reflect the dominant Communist ideology and hegemony in China (35). More recently, studies on HIV framing in Chinese media have focused on people living with HIV. For instance, Dan and Ren (36) content-analyzed Chinese news photos of people living with HIV and found that victim framing (i.e., emphasizing the weakness and illness of people living with HIV) was frequently used in these photos. Results from another content-analytical study (37) also found that victim framing was most frequently used in news texts about people living with HIV.

All of these studies [e.g., (33–35)] focused on how HIV issues were covered and presented in Chinese media, but none of them tested how such coverage influenced readers’ HIV beliefs. Moreover, the status of media consumption and the socio-cultural landscape in China have drastically changed since the early 2000s, as Chinese citizens gained wider access to the Internet, social media, and global information. To the best of our knowledge, scholars have yet to understand how HIV-related content in news media influences Chinese people’s HIV beliefs. Thus, the current study attempts to fill this gap by examining the framing effects of GSNs’ coverage on people’s HIV beliefs. In light of the study purposes and the results of previous studies on framing, we asked the following research questions:

RQ1: How do Chinese GSNs frame HIV in terms of content and valence?

RQ2: How do Chinese GSN readers describe HIV in terms of content and valence?

RQ3: Do GSN readers’ descriptions of HIV correspond with GSNs’ frames?

### 2.4. Moderators of framing effects

Several studies have examined framing effects by treating the media frames as the independent variable and framing outcomes (e.g., people’s opinions, issue interpretations, and attitudes) as the dependent variable [e.g., (23)]. However, such a simple proposition of “media frames lead to effects” overlooks the various moderating factors that could influence the process of framing (17). The current literature has examined several factors that could potentially enhance, limit, or eliminate framing effects (38).

The current literature examines two categories of moderating factors, namely individual and contextual factors. Individual-level factors include knowledge and personal values [e.g., (39, 40)]. For example, Shen and Edwards (40) conducted an experiment to examine how individual-level humanitarianism and individualism might moderate news framing effects in the context of welfare reform. They found that both individual-level core values interacted with news frames and influenced people’s thoughts and attitudes toward welfare reform. The research on contextual-level moderators examines source credibility, interpersonal communication, and issue importance (38, 41, 42). For example, Lecheler et al. (38) conducted two experiments and confirmed that “a high-importance issue yields no [framing] effects and a low-importance issue [yields] large [framing] effects” (p. 400). Interestingly, issue importance moderated the framing effects at both individual (i.e., important to someone personally) and contextual (i.e., hot or trendy issue in society) levels. However, there is sparse research on moderators of framing effects in China, and almost all previous research was conducted in Western democratic countries. Understanding of how various individual and contextual factors would moderate framing effects in China remains relatively rudimentary. It is reasonable to assume that these factors are specific to the context of the study and that the drastically different political, cultural, and individual factors in China might have unique impacts on news framing effects. Therefore, we proposed the following research question:

RQ4: What (a) individual and (b) contextual factors might explain the correspondence or discrepancies between Chinese GSNs’ framing and readers’ descriptions?

## 3. Three-phase mixed-methods approach

We conducted a three-phase mixed-methods study to examine the framing of HIV in Chinese GSNs and the framing effects. We conducted a deductive descriptive content analysis in the first phase, a cross-sectional online survey in the second phase, and in-depth expert interviews in the third phase. The three phases of the project followed a sequential order, with results from each previous phase informing the next. We used content analysis to answer the research question about how Chinese GSNs framed HIV (RQ1); we used the cross-sectional survey with an open-ended question to answer the research questions about how GSN readers describe HIV and the impacts of news framing (RQ2 & RQ3); and we used in-depth media expert interviews to explore the potential moderators that could have influenced the impact of framing (RQ4).

## 4. Phase 1: content analysis

### 4.1. Methods

In phase 1, we conducted a content analysis of HIV-related news articles (*n* = 78) published in two of the largest Chinese GSNs, *People’s Daily* and *Guangming Daily* (43). These two newspapers were selected based on their average circulation sizes and historical importance in mass communication in China. Both of these two newspapers are considered influential, elite, and prestigious among Chinese media (43, 44). *People’s Daily* is considered the mouthpiece of the Communist Party of China. This newspaper, for more than 70 years, “has adhered to the correct direction, and has actively promoted the party’s theory, line, principles and policies, and has actively promoted the central major decisions and plans, timely dissemination of information in various fields at home and abroad, in order that the Communist Party may unite and lead the people across the country to triumph in the revolution, construction, and reform” [*People’s Daily*, 2013, as cited in (45)]. *Guangming Daily* also represents the central Communist party leadership (43). Since its establishment in 1949, *Guangming Daily* has strictly followed the instructions and guidance from the leaders of the Chinese Communist Party (46). Another justification for selecting these two newspapers deals with availability. Although we did not have access to the digital or physical archives of GSNs in China, hard copies of *People’s Daily* and *Guangming Daily* could be found in public libraries in China. Thus, we visited public libraries in China and borrowed hard copies of these two newspapers. We examined all news articles from the two newspapers published between July 2018 and June 2019 (1 years’ worth of published news articles preceding the start of the project; the public libraries we visited had only 1 year’s worth of newspapers), and we identified the articles that were explicitly about HIV/AIDS. After identifying all the news articles, we conducted a deductive content analysis using seven frames that emerged from the previous literature (33–35). The seven frames were prevention methods, treatment options, causal interpretations, health outcomes, prevalence, moral evaluations, and political connections. The unit of analysis was each individual news article, and each article was coded for the presence of the seven frames (1 = *presence*, 0 = *absence*); one news article could have multiple frames. If a frame was present, we coded its valence (1 = *positive*, 2 = *negative*, 3 = *neutral*). The codebook is provided in the Supplementary material.

Two graduate students who were unaware of the research questions coded the news articles. These two coders were both native Chinese speakers and had a basic understanding of HIV issues. Coders were trained by the authors during multiple sessions with a set of 10 randomly selected example articles from the sample. These sessions lasted 90 minutes in total. Coders discussed the definition of each coding variable, coding criteria, and examples, and they pointed out any nuances. Inter-coder reliability was assessed with another 10 news articles from the sample, using Cohen’s Kappa (overall *κ* > 0.90). The authors and coders also discussed any disagreements until clarity and consensus were reached. Coding was completed in simplified Chinese without computer assistance.

### 4.2. Results

RQ1 asked how Chinese GSNs present HIV in terms of the presence and valence of frames. Table 1 presents the results for frame and valence coding. In terms of frame, out of the 78 articles, 50 (64.1%) presented the frame of HIV prevention; 49 (62.8%) presented the frame of HIV treatments; nine (11.5%) presented the frame of HIV causal interpretations; nine (11.5%) presented the frame of health outcomes; 15 (19.2%) presented the frame of HIV prevalence; nine (11.5%) presented the frame of moral evaluations; and 27 (34.6%) presented the frame of political connections related to HIV. In terms of valence, articles framed in terms of prevention, treatments, or moral evaluations tended to have either positive or neutral valence; articles framed in terms of health outcomes or prevalence tended to have either positive or negative valence; articles with a causal interpretations frame were overwhelmingly neutral; and articles with a political connection frame were overwhelmingly positive. Overall, the most common valences were positive (53.6%) or neutral (34.5%), with very few instances of negative valence (11.9%).

**Table 1:** Presence of frames and frame valence in newspaper articles.

Frames	Newspaper	Presence	Positive valence	Negative valence	Neutral valence
Prevention methods	Total	50 (64.1%)	26 (52.0%)	2 (4.0%)	22 (44.0%)
	GM	24 (57.1%)	12 (50.0%)	11 (45.8%)	1 (4.2%)
	PD	26 (72.2%)	14 (53.8%)	1 (3.9%)	11 (42.3%)
Treatment options	Total	49 (62.8%)	24 (49.0%)	4 (8.2%)	21 (42.8%)
	GM	23 (54.8%)	13 (56.5%)	1 (4.3%)	9 (39.2%)
	PD	26 (72.2%)	11 (42.3%)	3 (11.5%)	12 (46.2%)
Causal interpretations	Total	9 (11.5%)	0 (0%)	1 (11.1%)	8 (88.9%)
	GM	5 (11.9%)	0 (0%)	1 (20.0%)	4 (80.0%)
	PD	4 (11.1%)	0 (0%)	0 (0%)	4 (100%)
Health outcomes	Total	9 (11.5%)	4 (44.4%)	4 (44.4%)	1 (11.1%)
	GM	4 (9.5%)	1 (25.0%)	2 (50.0%)	1 (25.0%)
	PD	5 (13.9%)	3 (60.0%)	2 (40.0%)	0 (0%)
Prevalence	Total	15 (19.2%)	7 (46.7%)	7 (46.7%)	1 (6.7%)
	GM	4 (9.5%)	2 (50.0%)	2 (50.0%)	0 (0%)
	PD	11 (30.6%)	5 (45.5%)	5 (45.5.%)	1 (9%)
Moral evaluations	Total	9 (11.5%)	5 (55.6%)	1 (11.1%)	3 (33.3%)
	GM	4 (9.5%)	4 (100%)	0 (0%)	0 (0%)
	PD	5 (13.9%)	1 (20.0%)	1 (20.0%)	3 (60.0%)
Political connection	Total	27 (34.6%)	24 (88.9%)	1 (3.7%)	2 (7.4%)
	GM	11 (26.2%)	10 (90.9%)	1 (9.1%)	0 (0%)
	PD	16 (44.4%)	14 (87.5%)	0 (0%)	2 (12.5%)

GM, Guangming Daily; PD, People’s Daily. There was a total of 78 articles: 42 Guangming Daily articles and 36 People’s Daily articles. An article may have more than one frame. Percentages for frame presence are based on the total number of articles. Percentages for valence are based on the number of articles with each theme.

## 5. Phase 2: cross-sectional survey

### 5.1. Methods

After the content analysis, we conducted a cross-sectional survey with adult readers (*n* = 210) who subscribed to both *People’s Daily* and *Guangming Daily*. The research protocol was approved by the institutional review board of a large public university in the southeast region of the United States. Participants were recruited via Wen Juan Xing, one of the biggest online survey platforms in Mainland China. The platform functions like Qualtrics by assisting scholars and companies in survey distribution and participant recruitment. The survey assessed participants’ descriptions of HIV using an open-ended question. The open-ended responses were coded for the presence and valence of the frame using the same content analysis schemes used in the first phase of the study. Intercoder reliability was assessed using Cohen’s Kappa (overall *κ* > 0.90). The survey also assessed participants’ evaluations of the two GSNs, exposure to the two GSNs, and demographic variables.

### 5.2. Measures

#### 5.2.1. HIV description and beliefs

We used one open-ended question to assess participants’ descriptions of and beliefs about HIV. Specifically, the survey asked the participants how they would describe HIV by asking, “Imagine that you have a friend who does not know much about HIV. How would you describe HIV to your friend?” These data were coded for the presence of types of frame and frame valence.

#### 5.2.2. Newspaper evaluation

Seven items were used to assess participants’ evaluations of each of the two GSNs: accuracy, fairness, diversity, depth of information, sensationalism, currency, and refinement [five-point Likert scale, 1 = *strongly disagree*, 5 = *strongly agree*, (47)]. Higher scores indicate a more favorable evaluation. Example items include the following: “*People’s Daily* [*Guangming Daily*] provides accurate facts about an issue,” “*People’s Daily* [*Guangming Daily*] provides both positive and negative perspectives of an issue.” The items formed a reliable measure (*α* = 0.90). Overall, participants reported favorable evaluations of the newspapers (*M* = 3.90, *SD* = 0.48). There were no significant differences (*p* > 0.05) in evaluations between the two newspapers.

#### 5.2.3. Newspaper exposure

Exposure to the newspapers was measured using two items (i.e., “How often do you read the news articles from *People’s Daily* [*Guangming Daily*]?”) using a five-point Likert-type scale (1 = *rarely*, 5 = *very often*). Higher scores indicate a higher frequency of exposure. Participants reported a relatively high frequency of exposure (*M* = 3.93, *SD* = 0.95). There were no significant differences (*p* > 0.05) in exposure between the two newspapers.

### 5.3. Participants

About half of the participants were male (*n* = 107, 51%); the average age of the participants was 30.63 years (*SD* = 7.89). The large majority of the participants had a bachelor’s degree or higher (*n* = 193, 91.9%); the majority of participants either had a monthly income of 15 K Yuan or lower (approximately $2,150 USD; *n* = 72, 34.3%) or a monthly income between 15 and 35 K (approximately $2,150 to $5,500 USD; *n* = 101, 48.1%).

### 5.4. Results

RQ2 asked how Chinese GSN subscribers describe HIV in terms of the presence and valence of frames. Table 2 presents the results for frame and valence coding of participants’ descriptions of HIV. In terms of frame, out of the 210 open-ended descriptions of HIV, 41 (19.5%) presented the frame of HIV prevention; 36 (17.1%) presented the frame of HIV treatments; 83 (39.5%) presented the frame of HIV causal interpretations; 77 (36.7%) presented the frame of health outcomes; six (2.9%) presented the frame of HIV prevalence; 10 (4.8%) presented the frame of moral evaluations; and only one description (0.5%) presented the frame of political connections related to HIV. In terms of valence, descriptions that included frames of prevention or causal interpretations tended to be neutral in valence; descriptions that included frames of treatment, health outcomes, prevalence, and moral evaluations tended to be negative in valence; the one description that included a political connection frame was neutral in valence. Overall, the most common valences were neutral (54.7%) or negative (43.7%), with very few instances of positive valence (5.3%).

**Table 2:** Presence of frames and frame valence in participants’ descriptions (*n* = 210).

Frames	Presence	Positive valence	Negative valence	Neutral valence
Prevention methods	41 (19.5%)	2 (4.9%)	0 (0%)	39 (95.1%)
Treatment options	36 (17.1%)	5 (13.9%)	24 (66.7%)	7 (19.4%)
Causal interpretations	83 (39.5%)	0 (0%)	12 (14.5%)	71 (85.5%)
Health outcomes	77 (36.7%)	5 (6.5%)	57 (74.0%)	15 (19.5%)
Prevalence	6 (2.9%)	1 (16.7%)	4 (66.7%)	1 (16.7%)
Moral evaluations	10 (4.8%)	0 (0%)	10 (100%)	0 (0%)
Political connection	1 (0.5%)	0 (0%)	0 (0%)	1 (100%)

Participants’ descriptions may have more than one frame. Percentages for frame presence are based on the total number of descriptions. Percentages for valence are based on the number of descriptions with each theme.

To answer RQ3, we ran a series of chi-square tests with the count of the presence of each frame and the count of each valence of each frame. In terms of frames, results showed that there were significant discrepancies between newspapers’ framing and participants’ descriptions regarding the presence of all seven frames: HIV prevention (*χ^2^* = 52.29, *df* = 1, *p* < 0.001; Cramer’s *V* = 0.42, *p* < 0.001), HIV treatments (*χ^2^* = 57.04, *df* = 1, *p* < 0.001; Cramer’s *V* = 0.45, *p* < 0.001), causal interpretations (*χ^2^* = 20.49, *df* = 1, *p* < 0.001; Cramer’s *V* = 0.27, *p* < 0.001), health outcomes (*χ^2^* = 17.15, *df* = 1, *p* < 0.001; Cramer’s *V* = 0.24, *p* < 0.001), prevalence (*χ^2^* = 22.56, *df* = 1, *p* < 0.001; Cramer’s *V* = 0.28, *p* < 0.001), moral evaluations (*χ^2^* = 4.24, *df* = 1, *p* < 0.05; Cramer’s *V* = 0.12, *p* < 0.05), and political connections (*χ^2^* = 75.52, *df* = 1, *p* < 0.001; Cramer’s *V* = 0.51, *p* < 0.001). Examination of the observed and expected counts of each cell revealed that newspapers were more likely than participants to mention HIV in terms of prevention, treatments, prevalence, moral evaluations, and political connections, but they were less likely than participants to mention HIV in terms of causal interpretations and health outcomes.

In terms of frame valence, results also showed there were significant discrepancies between newspapers’ and participants’ descriptions: HIV prevention (*χ^2^* = 26.68, *df* = 2, *p* < 0.001; Cramer’s *V* = 0.54, *p* < 0.001), HIV treatments (*χ^2^* = 32.51, *df* = 2, *p* < 0.001; Cramer’s *V* = 0.62, *p* < 0.001), causal interpretations (*χ^2^* = 36.89, *df* = 2, *p* < 0.001; Cramer’s *V* = 0.59, *p* < 0.001), health outcomes (*χ^2^* = 12.39, *df* = 2, *p* < 0.01; Cramer’s *V* = 0.38, *p* < 0.01), moral evaluations (*χ^2^* = 15.35, *df* = 2, *p* < 0.001; Cramer’s *V* = 0.90, *p* < 0.001), and political connections (*χ^2^* = 8.64, *df* = 2, *p* < 0.05; Cramer’s *V* = 0.56, *p* < 0.05); there was no difference regarding the valence of prevalence frame (*χ^2^* = 1.79, *df* = 2, *p* = 0.41; Cramer’s *V* = 0.29, *p* = 0.41). Examination of the observed and expected counts of each cell revealed that newspapers were more likely than participants to use positive or neutral frames when mentioning HIV prevention, treatments, and political connections, but participants were more likely than newspapers to use negative or neutral frames when describing HIV causal interpretations, health outcomes, and moral evaluations. The full results of expected and observed counts for each frame valence and frames are presented in Table 3.

**Table 3:** Observed and expected counts for frames and frame valence.

Frames	Type	Presence	Positive valence	Negative valence	Neutral valence
Prevention methods	News	50 (24.6)	26 (15.4)	2 (1.1)	22 (33.5)
	Readers	41 (66.4)	2 (12.6)	0 (0.9)	39 (27.5)
Treatment options	News	49 (23.0)	24 (16.7)	4 (16.1)	21 (16.1)
	Readers	36 (62.0)	5 (12.3)	24 (11.9)	7 (11.9)
Causal interpretations	News	9 (24.9)	0 (0)	1 (1.3)	8 (7.7)
	Readers	83 (67.1)	0 (0)	12 (11.7)	71 (71.3)
Health outcomes	News	9 (23.3)	4 (0.9)	4 (6.4)	1 (1.7)
	Readers	77 (62.7)	5 (8.1)	57 (54.6)	15 (14.3)
Prevalence	News	15 (5.7)	7 (5.7)	7 (7.9)	1 (1.4)
	Readers	6 (15.3)	1 (2.3)	4 (3.1)	1 (0.6)
Moral evaluations	News	9 (5.1)	5 (2.4)	1 (5.2)	3 (1.6)
	Readers	10 (13.9)	0 (1.6)	10 (5.8)	0 (1.6)
Political connection	News	27 (7.6)	24 (23.1)	1 (1.0)	2 (2.9)
	Readers	1 (20.4)	0 (0.9)	0 (0)	1 (0.1)

Each observed count was followed by expected count within the parenthesis.

## 6. Expert interviews

As the results from the previous two phases indicated, there were significant discrepancies between the GSNs’ presentations and readers’ perceptions in both the presence and valence of each frame. Thus, we conducted in-depth interviews with media and public health experts who had extensive practical experience or research expertise in Chinese media and health information in China to seek their opinions on what factors could explain these discrepancies.

### 6.1. Methods

We recruited 15 Chinese national media and public health experts living in China or the United States. The interviewees were selected and recruited from the authors’ professional networks and further referrals. The interviews were in-depth and semi-structured, and they were all conducted *via* WeChat, the most frequently used communication app in China. We scheduled a time slot with each interviewee and placed each interviewee in a group chat (with one interviewee and two interviewers) before the interview started. Each interview lasted between 75 and 145 minutes, and the interviewees either typed written responses or sent a voice recording of their responses to answer our questions. We chose the group chat format because several interviewees raised privacy concerns (e.g., some of them prefer to not have their face or voice recorded). In a group chat on WeChat, interviewees could choose profile names and images that do not contain any personal information.

The interviews followed a semi-structured list of questions (see Supplementary material). We had five general categories of questions regarding GSNs’ roles, GSNs’ influences, the discrepancies in our findings, GSNs’ roles in HIV information dissemination, and their relationships with other media outlets in China. These general questions were usually followed by clarification and probing questions. Demographic information was also collected. We stopped interviewing when we reached data saturation and had sufficient information regarding the study aims (48). Interviews were transcribed verbatim. We conducted a qualitative descriptive analysis with the interview data (49) and presented the prominent themes and codes that emerged from the interviews. The quotes presented in the following results section were translated into English by two bilingual Chinese and English speakers. All names used are pseudonyms.

### 6.2. Participants

Ten of the 15 interviewees are female. Five of the interviewees currently live in the United States, and the rest of the interviewees live in China. All interviewees are Chinese. All interviewees had either a master’s degree or Ph.D. in relevant fields or were pursuing such a degree. Our interviewees strongly emphasized privacy concerns, especially with potential conversations regarding government agencies, governmental conduct, media censorship, and the LGBTQ community.

### 6.3. Results

RQ4 asked what individual or contextual factors might explain the discrepancies between Chinese GSNs’ framing of HIV and readers’ descriptions. The overall results suggested that the discrepancies between newspaper articles and readers’ descriptions could be explained by people’s attitudes toward GSNs and beliefs about HIV. Interviewees stated that these attitudes and beliefs could be further explained by the political and social environment, media ecology, historical framing, and cultural identities in China.

#### 6.3.1. People’s attitudes toward GSNs

Interviewees agreed that GSNs’ framing effects would be directly influenced by how Chinese people perceived government-sponsored news outlets. When asked about what readers and the general public thought of GSNs in China, most participants agreed that these newspapers were the “true voices of credibility and authority,” and they often spoke about the agenda of the Communist Party of China. These results aligned with our survey results in phase 2, where readers evaluated the two GSNs as credible and reliable.

However, our experts said these attitudes and beliefs could act as a double-edged sword in framing effects. On the one hand, interviewees mentioned the benefits of having a single authoritative voice in the time of national emergencies, such as GSNs’ coverage of the COVID-19 pandemic. Charlie said, “People finally started behaving themselves [during the COVID-19 pandemic] because of the serious tone from the state media and the strict community-based prevention methods these media promoted.” On the other hand, several interviewees said these images of “authority” and “the voice of the communist party” could also often backfire on framing effects. This could be especially true in non-emergency contexts such as HIV, when readers may not like an authoritative voice and subsequently not be influenced by the news framing of HIV. For example, Skyler observed that people more than often would disagree with some of the government’s actions or policies, because the ways of “unconditionally speaking along with the communist party [from the GSNs] leaves the impression that they are out of touch with the general public or lack of care of people’s true opinions.” This way of reporting might be perceived as “propaganda,” and such perceptions of “government propaganda” could override the informational, educational, and social values of the news. As Skyler put it, “While some of these newspapers’ coverage on HIV has strong educational values, people constantly feel these newspapers’ reports are not accurate because of the ‘hidden agenda.’ That consequentially diminishes the effectiveness of these coverages that GSNs set out to convey.”

In other cases, the credibility of all these news outlets could suffer because people start forming indifferent or even dismissive attitudes toward GSNs. Interviewees expressed that people could perceive that GSNs demonstrate a “lack of care of what people truly think or feel about an issue.” Timber explained this as “People felt [GSNs] had all the authority and they were ‘talking down to’ or ‘disciplining’ people. It is really hard to persuade others when the party being persuaded felt their perspectives were not even being acknowledged or accounted for.” Skyler summarized these effects as “The framing failed because people perceived state media as the higher-level voice that ‘talks down’ to people. Why would you listen to someone who ignores your view altogether?”

#### 6.3.2. Beliefs about HIV

Another factor that could explain the discrepancies was the topic itself. That is, the existing beliefs people have toward the issue being framed would determine how effective the framing is. One interviewee said, “It is about how people see HIV in China as much as it is about how people see GSNs.” Multiple interviewees pointed out that the populations and behaviors associated with HIV in China had been historically labeled as “questionable” or “immoral,” such as gay men, drug abuse, and casual sex. These populations and behaviors are still highly controversial and labeled as taboos in China. Interviewees used the term “谈艾色变” (which roughly translates to “face changes color when talking about AIDS”) to describe how most individuals in China react to HIV.

Given how stigmatized HIV is in China, people would treat news coverage of HIV very differently from other non-stigmatized issues. Dakota directly compared the framing of HIV versus the new property tax in Chinese GSNs. Although Chinese held unfavorable stands on both issues, Dakota said, “News coverage on a stigmatized issue, especially a multi-stigmatized issue like HIV, would generate automatic negative emotional reactions among people because these coverages directly challenge the pre-existing social stigmas they hold.” With a stigmatized issue, emotions and cognitive dissonance are more likely to be activated when encountering news information that might challenge pre-existing beliefs (50).

#### 6.3.3. Indirect background factors

Our results showed that the attitudes toward GSNs and people’s beliefs about HIV would influence the discrepancies between GSNs’ framing and readers’ descriptions. We further asked probing questions hoping to contextualize the origins of these attitudes and beliefs. Our interviewees stated four indirect factors that could explain these attitudes and beliefs further.

##### 6.3.3.1. Political and social environment

First, people have formed these attitudes toward GSNs because of China’s unique political and social environment. Several interviewees mentioned that the media influences were ultimately “in the hands of the communist party.” As Chris pointed out, “The party has the final says on all big matters; media do not function as the ‘check-and-balance mechanism or the whip’ to the government power like many Western countries. Instead, media often are tools to communicate the core social values.” Several interviewees stated the core value was “societal harmony,” and this value shaped how all media, including GSNs, selected their frames on many issues. One interviewee, Sage, said, “With relatively unbiased information, the most important task [for these news outlets] is to ensure political stability, societal harmony, and national development.” Although the information presented by these media outlets is considered to be credible and authoritative, many readers and the general public can sense the “hidden” party agenda. Although these values are not explicitly stated, they were constantly implicitly communicated. Chris stated, “The frames or angles given to an event, the extent of coverage given, and even the word choices are all implicit methods to communicate these values.” Consequently, people formed the perceptions that although GSNs were the voice of authority, they also were “tools for party agenda” that do not reflect people’s opinions.

##### 6.3.3.2. Media ecology

Intertwined with the political and social environment of China, the second contextual factor that could explain Chinese people’s attitudes toward GSNs was media ecology. Although Chinese GSNs operate as independent companies, the funding for these companies largely comes from the government. As several interviewees pointed out, these GSNs’ profit margins and survival did not depend on advertising sponsorship, size of circulation, or people’s ratings. That state of affairs would further enhance people’s attitudes that GSNs are mainly “the tools of party agenda,” and such attitudes would negatively impact framing effects as previously discussed.

Moreover, our interviewees mentioned that although GSNs might not be the most read news outlets in China, GSNs still are the core influencers of how other media outlets frame almost all issues. As Robin said, even though other news and media outlets are like “hundreds of blooming flowers,” government-sponsored news outlets still provide the “box that other media are allowed to play in.” Kai, a media professional in China, said, “These [GSNs] would never tell other news outlets what to cover or not cover; other news outlets must guess the ‘wind’ and make sure they do not write too far from how these GSNs cover a topic. Playing outside the box once or twice might be fine, but there could be negative consequences if you constantly go against the ‘wind.’” These unique influences on other media render the perceptions that GSNs could silo other voices and they could “talk down” to their audience. Although some might believe media outlets that more accurately reflect people’s opinions would have more journalistic influences, that is simply not the case in China.

##### 6.3.3.3. Historical framing of HIV

Third, several interviewees recalled the negative media frames and coverage of HIV in the 1990s and early 2000s. This coverage significantly contributed to and reinforced the stigmatizing HIV beliefs among most Chinese. For example, Charlie vividly recalled how Chinese media framed HIV as the product of “greedy people who sell blood in China” in the mid-1990s and the “unethical lifestyle of American pop culture” in the early 2000s. These historical frames created irrational fear of HIV and its spread, and these negative beliefs were “burned” in people’s minds for generations to come. The establishment of fear was so successful that these ingrained beliefs created automatic fear responses to HIV among Chinese. Taylor said, “People might be so stubborn and fearful about HIV, they might not even want to read any article about it. We all know [as scholars] that fear drives people to have irrational reactions, and HIV is one of them.” Given this negative historical framing, it might not be surprising to see how strongly people stigmatize HIV in China.

##### 6.3.3.4. Cultural identities

Lastly, some interviewees highlighted the potential impacts of the societal culture in China that could explain the social stigmas against HIV. Cultural factors indirectly influence the framing effects by reinforcing negative social stereotypes and stigmas against HIV in China. HIV might not be viewed as an individual or private matter in China; rather, many people could view it as a burden to the larger society. Avery used a metaphor and said, “It was like the bad students in your class when we were in grade school. Although their performances had nothing to do with you personally, many people would get mad because it made us look bad as a class.” Thus, whether framing can successfully change people’s beliefs (about HIV, at least) is not an individual-level issue but rather a community-based one. In addition, another cultural identity that could potentially influence the effectiveness of news framing is the strong “in-group–out-group” identity Chinese have. For example, Alex said, “People find multiple ways to establish their in-group identity or even superiority against the out-group. Having a socially stigmatized illness is a perfect opportunity for people to exclude someone from the inner group of the population.”

## 7. Discussion

The current study examined how Chinese GSNs framed HIV and the potential framing effects on readers’ HIV beliefs. The results indicated that there were significant discrepancies between news frames and readers’ descriptions of HIV. The results of the expert interviews suggested that these discrepancies could be explained by what people thought of GSNs and HIV in China, and these thoughts were associated with the political environment, media ecology, historical framing of HIV, and cultural identities in China.

### 7.1. The “simple” proposition of framing effects

**Figure 1:**
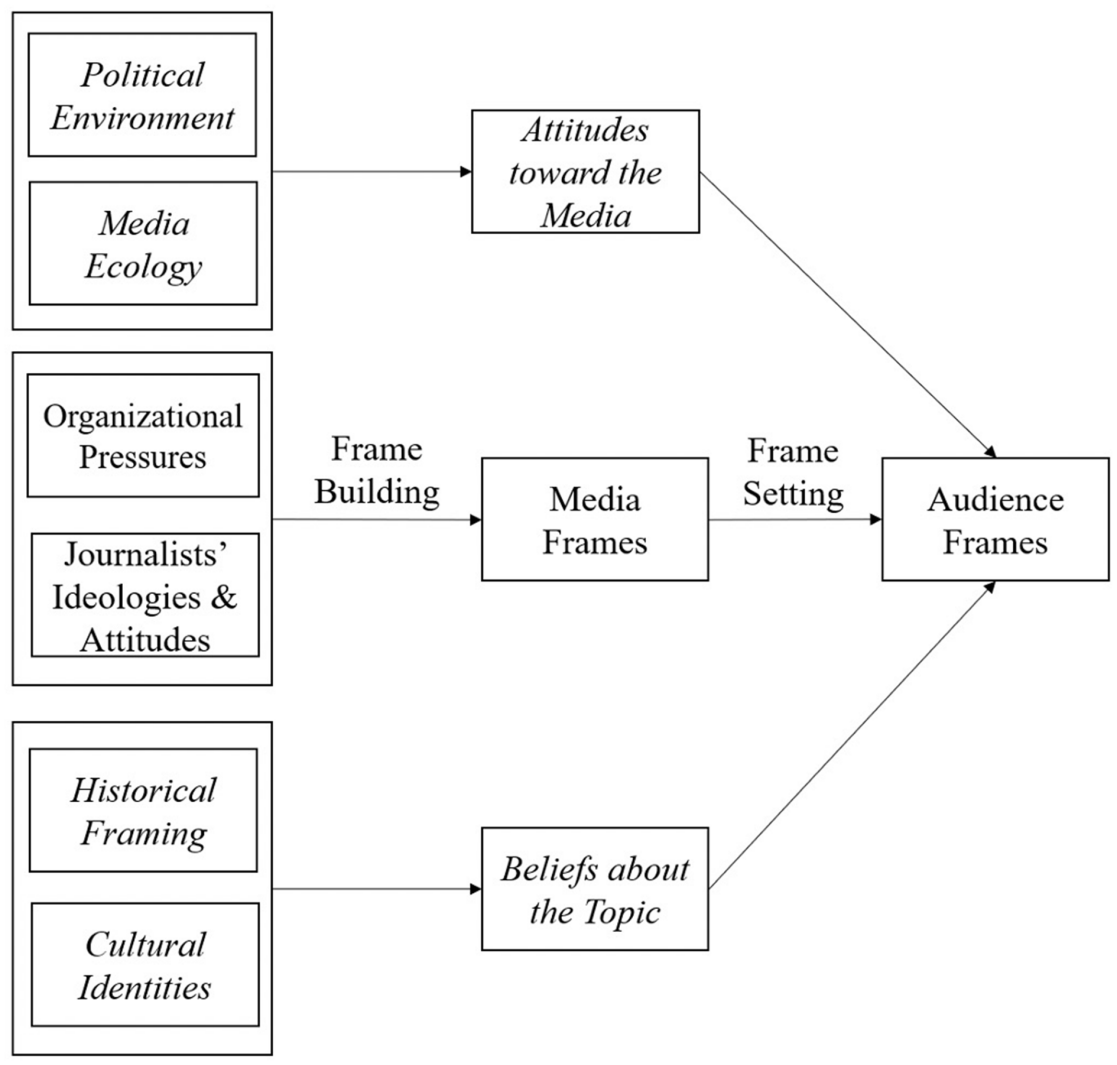
Framing process with moderators. Italics indicate additional specifications.

Many studies have examined framing exclusively in terms of the influences on audiences and assumed that there was a direct link between media frames and outcomes (18, 20). However, as previously discussed, a variety of factors could complicate such linear and direct framing effects [e.g., (38, 41, 42)]. In the following sections, we present some considerations that could potentially be useful when advancing the theoretical units and propositions of framing theory, particularly related to framing effects. We also present a visual representation of the framing effects with these moderating factors (see Figure 1).

### 7.2. Attitudes toward the media

The first moderating factor that could influence the potential framing effects is what the audience thinks of the media outlet. In the case of our study, Chinese GSN readers might find GSNs to be credible and authoritative but at the same time to be the “tools for the communist party agenda” without acknowledging people’s opinions and beliefs. These attitudes could lead to high resistance to the framing, especially when the topic being framed is a highly stigmatized one like HIV. The previous literature has documented how credible sources could “use a frame to alter the perceived importance of different considerations, and this, in turn, can change overall opinion,” whereas a noncredible source could not achieve the same [(41), p. 1059]. The previous research on attitudes toward media and its political party affiliation was primarily conducted in Western democratic countries [e.g., (51)]. In the case of political issue framing in Western countries, “citizens should be more likely to follow a frame if it is promoted by ‘their’ party” [(51), p. 630]. However, our results suggest that attitudes toward the media outlet are a sum of multifaceted factors that are rooted in a few contextual background factors, such as the political and social environment and media ecology.

### 7.3. Political and social environment

The current political and social environment in the country where the framing takes place could largely influence how people perceive certain types of media outlets. In the case of our study, our expert interviewees specifically spoke to how the Chinese Communist party sponsored and influenced almost all GSNs (at least people perceived so). The nature or attitudes toward such sponsorship left people with the perceptions that GSNs’ frames and news content often had a hidden political agenda or even served as “political propaganda.” Such perceptions that GSNs often have hidden political agenda consequentially reduced the relevance, educational value, and information value of the news coverage. The decrease in perceived news relevance might negatively impact people’s processing of HIV news, which subsequently led to failed frame setting. However, the literature on how the political and social environment changes people’s media outlet perceptions in China and the consequential news framing effects is quite lean. This might be partially due to the sensitive political environment in China and the perceptions of media censorship (52). We encourage future researchers to further explore the possibility of theoretical modifications related to political dynamics in framing research.

### 7.4. Media ecology

Media ecology refers to the environment in which multiple media compete and coexist, like the environmental ecology in which living beings coexist (53). Media ecology often uses the metaphor of evolution, where it “suggests new concepts and questions about media extinction, survival, and coevolution” [(53), p. 204]. The results of our study suggest that Chinese GSNs’ survival depends on the government instead of public popularity and persuasion effectiveness and that the GSNs’ frames reflected more of the government’s interests than the public interests. As the media content do not always exactly speak to the public agenda (12, 54), Chinese GSNs might be too “far out” from people’s perspectives in this case. Not leaning toward public interests at all may lead readers to perceive news content as irrelevant and allocate less attention and cognitive resources to the news information. This then may lead to a decrease in information processing and eventually a failure in the frame setting.

However, it is also critical to consider how different media compete and coexist with each other for survival and how those competitions influence potential framing effects. In the context of our study, Chinese GSNs have centralized power over other media and set a box (the frame of reference and the tone of coverage) for them. Such ecology with significant power differences between different media outlets is uncommon among Western democratic media, but the assumptions of media framing were arguably built on the assumption of equitable media competition (55). Thus, media ecology should be considered as an important theoretical unit to account for in future empirical examinations of framing effects.

### 7.5. Beliefs about the topic

The second direct moderating factor that could influence framing and potential framing effects is how people perceive the topic. Researchers should not assume that the potential framing effects related to coverage of HIV versus political elections would be the same even within the same political, media, and cultural context. Our interviewees discussed HIV as a multifaceted topic that intertwined with various political, social, cultural, and ethical stigmas, as HIV has often been labeled as the result of “immoral acts” in China. The nature of the topic is a stigmatized one in China. Stigmas are often associated with avoidance, intense negative reactions, and social distancing (56). Thus, readers who already have negative or stigmatizing HIV beliefs may utilize selective exposure or post-decision dissonance to justify and reinforce these beliefs (57). Future research should consider systematically examining how pre-existing beliefs toward stigmatized versus un-stigmatized topics moderate potential framing effects.

We also encourage future researchers to examine existing HIV information in the online media environment. Because of its accessibility and convenience, online information is a preferred source for people who are seeking health information about sensitive or stigmatized topics (58). Thus, the framing of HIV in online media may have an impact on information seekers’ perceptions of HIV. For people who are not motivated to seek information about HIV, passive encountering or routine patterns of exposure to online information (i.e., information scanning) also have the potential to influence their beliefs and knowledge about particular health topics (59).

### 7.6. Historical framing

Framing effects should be considered dynamically over time. Our interviewees discussed the impacts of negative historical coverage on HIV in China and speculated that time would be needed to “correct” that historical framing. Chinese government-sponsored media framed HIV as an “immoral disease” in the mid-1990s and the early 2000s, a time period during which government-sponsored media had profound impacts in shaping public opinions (60). Such historical coverage of HIV resulted in deeply ingrained negative HIV beliefs, and public education regarding HIV has not been implemented until fairly recently. Thus, the stigmatized HIV beliefs might have been passed on through generations, which could still influence how people see HIV in China today. In addition, changing beliefs regarding an issue is more difficult than reinforcing pre-existing beliefs or shaping beliefs with no prior formed position, as would have been the case initially with HIV (61). As one of our interviewees, Charlie, pointed out, scholars need to consider “the state of the canvas that the media is painting on” when studying media framing effects. We encourage scholars to investigate how to mitigate the effects of historical framing on stigmatized topics.

### 7.7. Cultural identities

Pre-existing beliefs toward the topic being framed could also be influenced by shared cultural identities. Van Gorp (62) suggests that frames are part of the culture and that culture inevitably influences the production and interpretation of the news. In the case of our study, we found that both collectivistic and strong “in-group–out-group” identities could potentially reinforce the stigmatized views of HIV, which then makes the GSNs’ framing less effective.

China, in general, has been considered a collective society. How this cultural identity could have negatively impacted the potential framing effects on HIV might be 3-fold. First, when it comes to stigmatized illnesses, the public could perceive people infected as a shame and burden to the group and the country. Second, because of the stigma and stereotypes associated with HIV (e.g., HIV is a result of deviant behaviors and a violation of moral norms), people living with it would no longer be perceived as an in-group member, as the deviance or violation of group norms often warrants exclusion (63, 64). Collectivistic members tend to care less about out-group members. Third, because HIV is stereotypically perceived as highly contagious and deadly, in-group members need to protect the well-being of other in-group members and further exclude people who live with HIV. Relevant cultural identities are topic-specific and should be placed within the context of existing beliefs.

### 7.8. Practical implications

The current study has some practical implications for HIV information dissemination and social campaigns in China. Specifically, the interviews with media and public health experts indicated that newspaper readers might feel that GSNs were not catering to the readers’ interests and perspectives and that GSNs were “talking down to” or “disciplining” the readers, which subsequently led to the formation of indifferent attitudes toward GSNs and failed frame setting (see section 6.3.1 for more details). Thus, we suggest that news media may want to include emotion-based coverage when reporting HIV issues. This type of coverage might better connect with the audience’s lives and emotions, which in turn may help the audience see and understand HIV issues and HIV patients through the lens of “people’s stories.” Similarly, journalists at GSNs may need to find a new way to balance the government agenda and public interests. Although they may be obligated or required to report news content in a way that ensures political stability and societal harmony, journalists may want to try to incorporate some content that is relevant to people’s lives. For instance, when reporting new policies on HIV medicines, journalists can emphasize the hard work the government has done but also detail the benefits or desirable outcomes on actual people’s lives, catering to both the government’s and the public’s interests. Moreover, several interviewees said that it would be more effective to implement an entertainment-education approach, such as movies or short clips about people living with HIV. This approach has been historically more effective in changing how people perceive minority groups in China because it is “more connected to life” and calls for “emotional reactions than cold statistics” (65).

### 7.9. Limitations and future research

There are three primary limitations in the current study. First, the content analysis focused on only two of the major GSNs in China. We encourage future researchers to examine other GSNs and privately-owned media outlets to gain a more comprehensive view of how news media have framed HIV issues in China. Second, we conducted expert interviews to help explain the discrepancy between the content analysis and survey findings instead of interviewing readers of the GSNs. We believe that interviewing experts offered more insightful views on this issue regarding the discrepancies between news frames and public frames. However, future research could conduct qualitative research with readers regarding these discrepancies to get the other side of the story. Third, due to privacy concerns, we did not use real-time video calls when conducting expert interviews. Video or face-to-face interviewing allows the observation of facial expression and body language, which may enhance data richness (66).

## 8. Conclusion

Through a three-phase mixed-methods study, we examined HIV news frames and the potential framing effects from Chinese GSNs. The results indicated that there were significant differences between GSNs’ frames and readers’ HIV beliefs. Our expert interviewees suggested that the discrepancies could be explained by people’s attitudes toward GSNs and beliefs about HIV in China, which could be further explained by the political environment, media ecology, historical framing, and cultural identities. We hope our results offer some insights into how media framing could impact public health issues, especially in the intriguing geopolitical context and media landscape of China.

## Data availability statement

The raw data supporting the conclusions of this article will be made available by the authors, without undue reservation.

## Ethics statement

The studies involving human participants were reviewed and approved by Institutional Review Board, Office of Research Integrity, University of Kentucky. The patients/participants provided their written informed consent to participate in this study.

## Author contributions

TC and MD contributed to conception and design of the study, statistical analysis, and manuscript writing. NH contributed to the design of the study and manuscript revision. All authors contributed to the article and approved the submitted version.

## Conflict of interest

The authors declare that the research was conducted in the absence of any commercial or financial relationships that could be construed as a potential conflict of interest.

## Publisher’s note

All claims expressed in this article are solely those of the authors and do not necessarily represent those of their affiliated organizations, or those of the publisher, the editors and the reviewers. Any product that may be evaluated in this article, or claim that may be made by its manufacturer, is not guaranteed or endorsed by the publisher.
