# Supplementary material for: Understanding the audience in framing research: empirical evidence from three studies examining HIV framing in China
Source: Front Public Health. 2023 Aug 17;11:1172020. doi: 10.3389/fpubh.2023.1172020 (PMC10469626; doi:10.3389/fpubh.2023.1172020)
Supplement: Supplementary file 1 [file Table_1.DOCX]

**Frame and Frame Valence Codebook**

| **Variables** | **Definition** | **Code** | **Examples** |
| --- | --- | --- | --- |
| Prevention Methods | Description of HIV prevention methods, including condom use, PrEP, abstinence, and not sharing needles | Presence: 1  Absence: 0 |  |
| Prevention Methods_Valence | Outlooks, attitudes, evaluations, language use, tone in the description | Positive: 1  Negative: 2  Neutral: 3  Absence of Description: 0 | Positive: One HIV vaccine passed early human trials. It can induce immune responses and is safe and effective.  Negative: HIV prevention is complicated because there is a window period. Some infected people cannot be detected by current testing methods at the initial stage of infection, which will cause their donated blood to be used as normal blood for clinical use. This factor cannot be controlled or prevented in the current stage.  Neutral: HIV prevention methods include PrEP. |
| Treatment Options | Description of treatment options, including medicines, therapies | Presence: 1  Absence: 0 |  |
| Treatment Options_Valence | Outlooks, attitudes, evaluations, language use, tone in the description | Positive：1  Negative：2  Neutral：3  Absence of Description: 0 | Positive: We have a new treatment plan, which has opened the door of hope for the AIDs treatment and can be regarded as an epoch-making milestone.  Negative: There is currently no cure for AIDS；HIV treatment is complicated.  Neutral: Cocktail therapy is often used in treating HIV infection. Cocktail therapy refers to the combined use of protease inhibitors and multiple antiviral drugs to reduce the resistance caused by a single drug. |
| Casual interpretation | Description of risk factors, cause, origin, and transition of HIV | Presence: 1  Absence: 0 |  |
| Causal interpretations _ valence | Outlooks, attitudes, evaluations, language use, tone in the description | Positive: 1  Negative: 2  Neutral: 3  Absence of Description: 0 | Positive: there is no positive-valence causal interpretations frame in the sample.  Negative: “Bad” sex is the most common way of getting infected; AIDS is transmitted through “improper” sex.  Neutral: HIV is transmitted through the exchange of body fluids such as blood and semen. |
| Health Outcomes | Description of HIV health outcome or health outcome of HIV-infected people | Presence: 1  Absence: 0 |  |
| Health Outcomes_Valence | Outlooks, attitudes, evaluations, language use, tone in the description | Positive: 1  Negative: 2  Neutral: 3  Absence of Description: 0 | Positive: There is no need to worry too much. Take medicine as prescribed, HIV patients can still live long and have a normal life.  Negative: Getting infected means death; there is no chance of surviving.  Neutral: Without treatment, the life expectancy after the final stage is 12 to 18 months. |
| Prevalence | Description of HIV prevalence and incidence | Presence: 1  Absence: 0 |  |
| Prevalence_Valence | Outlooks, attitudes, evaluations, language use, tone in the description | Positive: 1  Negative: 2  Neutral: 3  Absence of Description: 0 | Positive: The AIDS prevalence in our country is low; HIV infections have decreased by 5.86% as compared to the same period last year, indicating a relatively optimistic situation.  Negative: HIV situation is severe and serious in Guangdong Province. From January to October this year, Guangdong reported a total of 7,326 new cases, an increase of 20% as compared to the same period last year.  Neutral: AIDS is a globally prevalent disease. |
| Moral Evaluations | Descriptions of moral issues related to HIV, including judgement, evaluation, stereotype, and stigma | Presence: 1  Absence: 0 |  |
| Moral Evaluations_Valence | Outlooks, attitudes, evaluations, language use, tone in the description | Positive: 1  Negative: 2  Neutral: 3  Absence of Description: 0 | Positive: Don’t treat HIV patients and the virus as scourges; The whole society needs to have empathy and acceptance for HIV patients  Negative: Getting infected is a result of conducting immoral acts.  Neutral: Social discrimination hinders people’s understanding of AIDS |
| Political Connection | Description of policies, regulations, and laws related to HIV | Presence: 1  Absence: 0 |  |
| Political Connection_Valecne | Outlooks, attitudes, evaluations, language use, tone in the description | Positive: 1  Negative: 2  Neutral: 3  Absence of Description: 0 | Positive: To help HIV patients to the maximum extent, our country has issued a series of policies regarding AIDS drug supply, medical treatment, care and assistance, etc.  Negative: These two HIV patients are happy together and want to get married. However, they are prohibited from getting married because the current Marriage Law states that HIV is a “marriage-inappropriate” disease.  Neutral: We need to establish laws regarding people intentionally infecting someone with HIV. |

*Please note all the examples were from the sample and this codebook was manually translated from simplified Chinese to English by two bilingual experts.

**Interview Protocols**

In the previous study, we first summarized the articles on HIV from two major government-sponsored newspapers (People’s Daily and Guangming Daily). After that, we asked the readers of these two newspapers to freely express their views on HIV. We compared the two sets of data and found some drastic differences. In summary, the news articles premilitary focused on the comprehensive knowledge and positive aspects of HIV, but the readers’ views on HIV were relatively negative and stigmatizing. In other words, the government-sponsored newspaper uses science communication in politically correct views, while the readers’ reactions were filled with negativity and stigmas. For example, the government-sponsored newspapers vigorously promoted the country’s various free HIV medical programs and the correct protection methods for HIV. However, newspaper readers still regarded HIV infections as a moral problem (a disease caused by people’s own behavior) and perceived HIV patients as a social burden. In addition, positive information related to prevention and treatment will often appear in these news articles, but the public perceived HIV as “uncurable” and a “lifestyle disorder.” The data showed that these readers were all highly educated and had a healthy income. They regularly read these government-sponsored newspapers and had favorable evaluations of these newspapers. This leads us to wonder as to why there was such a big gap between the content of the government-sponsored newspaper and the reader’s opinion. What factors or moderators could have caused these gaps?

Because you have extensive knowledge of Chinese culture, media, politics, and the overall societal structure, we have recruited you to help us solve the questions mentioned above. In this interview, we will ask a series of questions, and I hope you can speak freely. In order to ensure anonymity and personal privacy, we will not collect your name or any other identifiable personal information. If you do not want to answer a specific question, you can choose to skip it. In order to protect your privacy to the utmost extent, during the discussion, we hope that you can express your views by typing out written messages. You can also directly use the voice and then convert it into written messages to send out (WeChat has this function already). Do you have any questions about the above text? If not, we will start this interview.

**The government-sponsored newspapers’ roles**

1. How do you think news media consumers/readers perceive government-sponsored newspapers?
2. How do you think the public perceives government-sponsored newspapers?
3. What roles do government-sponsored newspapers have among Chinese media outlets?

**Government-sponsored newspapers’ influences and the relationships with other media outlets in China**

1. How do government-sponsored newspapers influence people’s opinions?
2. To what degree do government-sponsored newspapers influence people’s opinions?
3. How do government-sponsored newspapers, directly and indirectly, influence how other news and media outlets in China cover a topic?

**The discrepancies in our findings**

1. Our research results showed that the content of newspaper reports and readers’ opinions were significantly different. Why do you think this might be the case?
2. What factors or moderators might cause these discrepancies?
3. How do government-sponsored newspapers influence these discrepancies?
4. How does the topic of HIV influence these discrepancies?

**Government-sponsored newspapers’ roles in HIV information dissemination**

1. What are the roles of government-sponsored newspapers in helping the public understand HIV?
2. What should the roles of government-sponsored newspapers in helping the public understand HIV?
3. How do we improve understanding about HIV through mass and new media?

*Please note this guide was manually translated from simplified Chinese to English by two bilingual experts.
